# Oligomeric amyloid-β induces early and widespread changes to the proteome in human iPSC-derived neurons

**DOI:** 10.1038/s41598-020-63398-6

**Published:** 2020-04-16

**Authors:** Christopher Sackmann, Martin Hallbeck

**Affiliations:** Department of Clinical Pathology and Department of Biomedical and Clinical Sciences, Linköping University, Linköping, Sweden

**Keywords:** Cellular neuroscience, Alzheimer's disease, Neurodegeneration, Molecular neuroscience, Neurodegeneration

## Abstract

Alzheimer’s disease (AD) is the most common form of dementia globally and is characterized by aberrant accumulations of amyloid-beta (Aβ) and tau proteins. Oligomeric forms of these proteins are believed to be most relevant to disease progression, with oligomeric amyloid-β (oAβ) particularly implicated in AD. oAβ pathology spreads among interconnected brain regions, but how oAβ induces pathology in these previously unaffected neurons requires further study. Here, we use well characterized iPSC-derived human neurons to study the early changes to the proteome and phosphoproteome after 24 h exposure to oAβ 1-42. Using nLC-MS/MS and label-free quantification, we identified several proteins that are differentially regulated in response to acute oAβ challenge. At this early timepoint, oAβ induced the decrease of TDP-43, heterogeneous nuclear ribonucleoproteins (hnRNPs), and coatomer complex I (COPI) proteins. Conversely, increases were observed in 20 S proteasome subunits and vesicle associated proteins VAMP1/2, as well as the differential phosphorylation of tau at serine 208. These changes show that there are widespread alterations to the neuronal proteome within 24 h of oAβ uptake, including proteins previously not shown to be related to neurodegeneration. This study provides new targets for the further study of early mediators of AD pathogenesis.

## Introduction

Alzheimer’s disease (AD) is the most prevalent form of dementia globally and is expected to grow substantially along with the aging global population. Much of the research in this field has focused on the pathological hallmarks of AD, amyloid-β (Aβ) and tau, but the way in which these mediators initiate neuronal pathogenesis at the molecular level requires further understanding. An important consideration in the study of AD is the different properties of Aβ assemblies: monomers, oligomers and fibrils. In particular, there is growing evidence to suggest that low molecular weight Aβ oligomers (oAβ) and protofibrils seed the aggregation of naïve Aβ, confer neurotoxicity, and also correlate to cognitive decline, as reviewed in^1–4^. Much of our current understanding of AD comes from the study of long-term exposure to Aβ in animal models, which has provided valuable insight into the effects of chronic exposure to Aβ, but often overlook the initial steps involved in pathogenesis. In order to further our understanding of the acute effects of exposure to oAβ, we challenged human iPSC-derived neurons with oAβ for 24 h and examined the changes to the proteome and phosphoproteome elicited by this acute oAβ treatment. In this way, we aim to elucidate some of the early effects elicited by oAβ upon neurons.

During AD, widespread changes occur in the proteome, including the up- and downregulation of disease-related proteins as well as the post-translational modification (PTM) of proteins. PTMs such as phosphorylation greatly affect the function of proteins and are well known to be important for the pathogenesis of neurodegenerative disorders, where detection of phosphorylated proteins are used to diagnose the severity of pathology^5,6^. Other types of PTMs include glycosylation, nitration, ubiquitination, truncation, methylation, and acetylation, among others. PTMs have significant influence upon the protein secondary and tertiary structure, which in turn impact its function, and importantly to neurodegeneration, its aggregation potential. During AD, PTMs of the tau protein are thought to lead to alterations in its ability to bind and stabilize microtubules and these PTMs also contribute to its misfolding and aggregation. The homeostasis of tau phosphorylation is delicately regulated by major kinases and phosphatases including the Casein Kinase (CK) and Glycogen Synthase Kinase 3 families (GSK3), as well as the Protein Phosphatase family (PP). In AD, CK1ε and GSK3β are increased, while PP2A and PP5 are decreased, contributing to the hyperphosphorylation of tau^7,8^. The association between tau and Aβ and their role in the initiation and driving of AD related neurodegeneration is a major topic of debate. Previous studies have demonstrated that Aβ can induce tau PTMs, leading to erroneous distribution of tau and neurotoxicity^9,10^. Others have shown the ability of oligomeric Aβ to bind tau and promote its fibrillization^11–13^, indicating a close relationship between Aβ and tau in AD pathogenesis.

While targeted interaction studies like those involving Aβ and tau have been the subject of many studies, unbiased proteomics approaches to understanding changes to the proteome in response to Aβ are lacking, leaving a significant gap in the understanding of how oAβ induces pathology in naïve human neurons. In this study, we use two well characterized human neuronal cellular models derived from human iPSCs: one harbouring wild-type amyloid precursor protein (APP; AF22) and another with the London APP (V717I) mutation (ADP2)^14–21^. Using LC-MS/MS and label-free quantification, we investigated the early changes to the proteome and phosphoproteome after neurons were challenged with oAβ, simulating a cellular environment comparable to early oAβ pathology in AD^22–25^. Using this approach, we identified expression alterations in response to oAβ challenge, including the mRNA processing proteins TDP-43 and hnRNP-F/K, vesicle trafficking proteins VAMP1/2 and coatomer subunits delta/gamma-1, among others. Both WT APP and V717I APP neurons displayed a marked decrease in coatomer complex I (COPI) subunit expression, indicating that intracellular retrograde transport pathways are greatly affected by oAβ. Interestingly, we also observed the differential phosphorylation of tau at serine 208 in response to the oAβ challenge. The alterations described in this study provide insight toward the early effects mediated by oAβ, adding new understanding of AD pathogenesis and identifying potential novel targets for early intervention in AD and other amyloidopathies.

## Results

### Protein identification by nLC-MS/MS

**Figure 1:**
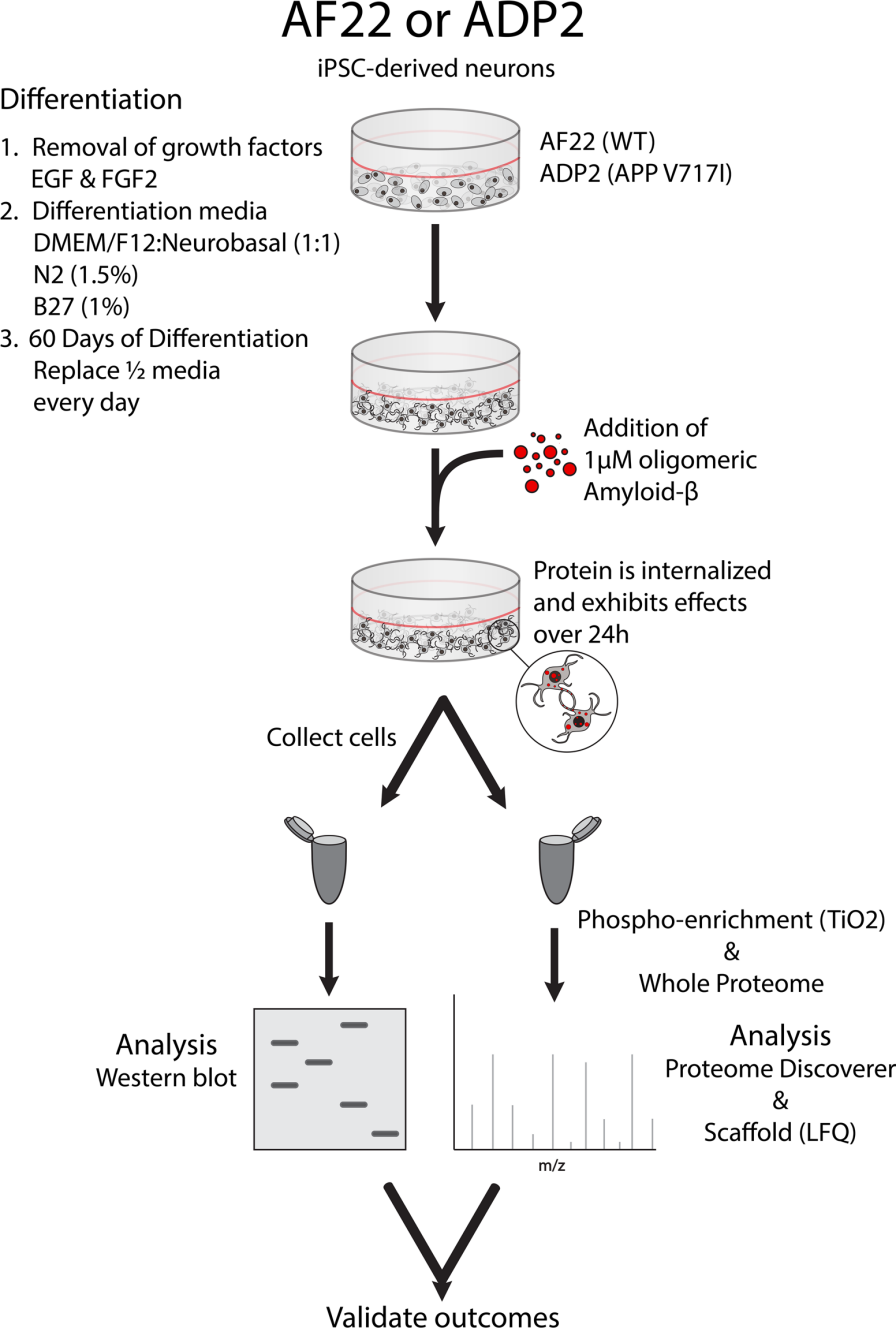
Depiction of cell treatment models. iPSC-derived neurons are first differentiated for 60 days, then treated with 1 µM oligomeric amyloid-beta for 24 h. Neurons are collected and used for Western blot, or with nLC-MS/MS (whole proteome analysis and phosphoenrichment). Quantification is performed by densitometry (Western blot) and by label-free quantification (LFQ) using Scaffold proteomics software. Fold-change values were compared between both methods to validate the LFQ method.

To obtain neuronal phenotypes in our human cellular model, long-term neuroepithelial-like stem cells (ltNES) were differentiated for 60 days prior to addition of 1 µM oligomeric amyloid-β (oAβ). This differentiation process results in cells with phenotypes indicative of functional neurons, including mature electrophysiological activity and the expression of neuronal markers NeuN and β-III tubulin. Differentiated cells were predominantly neurons as determined by their expression of the neuronal nuclear marker NeuN (AF22 = 78.5 ± 5.4%, ADP2 = 84.5 ± 9.0%), consistent with previous studies using these ltNES cells (Supplementary Fig. S1)^14–21^. After 24 h incubation with the oAβ, cells were collected, and proteins extracted for whole proteome or phosphoproteome analysis by nLC-MS/MS (summarized in Fig. 1). The raw data were analyzed using the Sequest HT algorithm in Proteome Discoverer and exported to Scaffold to perform a comparative differential analysis, employing Student’s t-test using normalized Total TIC. These analyses were performed in 3 datasets: AF22 (Wild-type APP; n = 16 control, n = 14 oAβ treated), AF22 phosphoenriched (PPE) with TiO_2_ (n = 11 control, n = 8 oAβ treated) and ADP2 (V717I APP mutation; n = 7 control, n = 7 oAβ treated). These analyses resulted in the identification of 2253 proteins (1627 protein groups; AF22), 1724 proteins (1315 protein groups; ADP2) and 577 proteins (498 protein groups, AF22 PPE), respectively.

### Protein expression in human iPSC-derived neurons are significantly altered in response to oAβ

In the AF22 proteome, 42 proteins were found to be significantly (p < 0.05) up- or downregulated (Table 1). Several of these were of particular relevance to cellular pathways implicated in AD, including significant upregulations in vesicle-associated protein VAMP2 (fold change 1.5, p = 0.036), the Aβ binding protein SGTA (small glutamine-rich tetratricopeptide repeat-containing protein alpha; fold change 1.9, p = 0.018) and the AD-associated, CNS development and maturation protein DPYL5 (Dihydropyrimidinase-related protein 5, AKA CRMP5, fold change 1.4, p = 0.047). In response to the oAβ challenge, there were also significant downregulations observed in several proteins, including the neurodegenerative disease (ND)-related protein TDP-43 (fold change 0.4, p = 0.038) and COPI retrograde trafficking protein coatomer subunit delta (fold change 0.2, p = 0.026) were observed in response to oAβ. Next, we queried this dataset for several possible PTMs, which identified 10 proteins with significantly altered PTM expression in response to oAβ, including acetylation of 14-3-3ε (fold change 0.3, p = 0.0097), methylation of tubulin beta-3 chain (fold change 0.07, p = 0.036) and phosphorylation of stathmin (fold change 2.1, p = 0.046) (Supplementary Table S2). We have also provided a curated list of proteins of interest with relevance to the studies of NDs, including tau (fold change 1.8, p = 0.25) and protein phosphatase 5 (PP5; fold change 0.2, p = 0.11), α-synuclein (fold change 1.9, p = 0.4), among others (Supplementary Table S3).

**Table 1:** Significantly up- and downregulated proteins in the proteome of AF22 cells resulting from 24 h oAβ treatment.

Identified Proteins	Protein ID	AF22 T-Test (p-value)	AF22 Fold Change	ADP2 T-Test (p-value)	ADP2 Fold Change
Amyloid beta A4 protein	A4_HUMAN	0.0035	3.3	0.067	17
Aspartate aminotransferase, cytoplasmic	AATC_HUMAN	0.015	1.8	0.58	1.5
Cytoplasmic aconitate hydratase	ACOC_HUMAN	0.049	0.3	0.19	0
Proteasomal ubiquitin receptor ADRM1	ADRM1_HUMAN	0.035	3.8	0.13	INF
Actin-related protein 2/3 complex subunit 3	ARPC3_HUMAN	0.032	0.3	0.18	2.8
Cluster of Isoform Beta of Apoptosis regulator BAX	BAX_HUMAN	0.015	0.4	0.65	1.2
Bleomycin hydrolase	BLMH_HUMAN	0.04	0.6	ND	ND
BRI3-binding protein	BRI3B_HUMAN	0.011	2.6	ND	ND
60 kDa heat shock protein, mitochondrial	CH60_HUMAN	0.039	1.3	0.66	1.1
Cluster of Isoform 2 of Clathrin heavy chain 1	CLH1_HUMAN	0.041	0.7	0.52	1.2
Cluster of Cofilin-1	COF1_HUMAN	0.024	0.7	0.97	1
Coatomer subunit delta	COPD_HUMAN	0.026	0.2	0.88	1.2
Casein kinase II subunit alpha	CSK21_HUMAN	0.029	0.5	0.54	1.5
Dihydropyrimidinase-related protein 5	DPYL5_HUMAN	0.047	1.4	0.3	1.4
Cluster of Spliceosome RNA helicase DDX39B	DX39B_HUMAN	0.022	0.5	0.58	0.7
Cluster of Isoform 2 of Elongation factor 1-delta	EF1D_HUMAN	0.038	1.6	1	1
Cluster of Isoform 2 of Histone deacetylase 2	HDAC2_HUMAN	0.027	0.4	0.21	0.05
Heterogeneous nuclear ribonucleoprotein F	HNRPF_HUMAN	0.0087	0.5	0.78	0.9
LIM and SH3 domain protein 1	LASP1_HUMAN	0.038	2.8	0.17	3.6
Leucine-rich PPR motif-containing protein, mitochondrial	LPPRC_HUMAN	0.017	0.07	0.43	0.4
Microtubule-associated protein RP/EB family member 1	MARE1_HUMAN	0.015	2	0.9	1.1
Isoform 2 of RNA-binding protein Musashi homolog 2	MSI2H_HUMAN	0.037	0.5	0.47	0.2
Metallothionein-3	MT3_HUMAN	0.033	9.8	ND	ND
Isoform 2 of NADH dehydrogenase 1 alpha subcomplex subunit 11	NDUAB_HUMAN	0.015	0.3	0.34	INF
Isoform 2 of Nuclear mitotic apparatus protein 1	NUMA1_HUMAN	0.03	3.8	ND	ND
Isoform 2 of GPI transamidase component PIG-S	PIGS_HUMAN	0.042	0.5	0.91	0.9
Isoform 2 of Proteasome subunit alpha type-3	PSA3_HUMAN	0.038	1.8	0.13	4
Cluster of 26 S proteasome non-ATPase regulatory subunit 4	PSMD4_HUMAN	0.046	2.6	0.21	3.3
Multifunctional protein ADE2	PUR6_HUMAN	0.04	0.5	0.99	1
Cluster of 60 S ribosomal protein L26	RL26_HUMAN	0.018	0.3	0.22	2.4
Small glutamine-rich tetratricopeptide repeat-containing protein alpha	SGTA_HUMAN	0.018	1.9	0.31	1.5
Synaptogyrin-3	SNG3_HUMAN	0.036	2.1	0.32	2.6
Signal recognition particle subunit SRP72	SRP72_HUMAN	0.045	0	0.34	0
Translocon-associated protein subunit delta	SSRD_HUMAN	0.022	0.5	0.61	0.8
Phenylalanine–tRNA ligase alpha subunit	SYFA_HUMAN	0.037	0.5	0.8	0.8
TAR DNA-binding protein 43	TADBP_HUMAN	0.038	0.4	0.64	0.8
Transformer-2 protein homolog beta	TRA2B_HUMAN	0.047	0.4	0.27	0.5
Cluster of Isoform 3 of Thioredoxin reductase 1, cytoplasmic	TRXR1_HUMAN	0.041	0.4	0.11	3.8
Isoform 3 of 116 kDa U5 small nuclear ribonucleoprotein component	U5S1_HUMAN	0.03	0.2	0.52	0.5
Isoform 3 of Vesicle-associated membrane protein 1	VAMP1_HUMAN	0.039	87	0.99	1
Cluster of Vesicle-associated membrane protein 2	VAMP2_HUMAN	0.036	1.5	0.29	1.5

The provided table depicts proteins with significant changes in expression (p < 0.05, T-test with Benjamini-Hochberg correction) in cells challenged with oAβ relative to untreated cells (i.e. AF22 + oAβ relative to AF22 control). AF22: n = 16 (control), n = 14 (oAβ treated). Data represent analysis of whole proteome preparations. ADP2 columns provided for comparison. ADP2: n = 7 (control), n = 7 (oAβ treated). ND = Not detected.

In order to confirm the validity of the LFQ values determined by normalized Total TIC, we analyzed the proteins from AF22 neurons by Western blotting and compared the LFQ outcomes to those determined by densitometry (Table 2). We examined 6 proteins by Western blot (Supplementary Figs. S2 and S3) and found that the fold change values and significance obtained by both methods (LFQ and Western blot) gave similar results, providing confidence in the data obtained by this method.

**Table 2:** Verification of the validity of quantifications by Normalized Total TIC (MS/MS LFQ).

Protein	Protein ID	Fold Change (MS/MS)	p-value (MS/MS)	Fold Change (WB)	p-value (WB)
Calreticulin	CALR_HUMAN	1	0.89	1.21	0.67
Ras-related protein Rab-7a	RAB7A_HUMAN	1	0.89	0.98	0.97
TAR DNA-binding protein 43	TADBP_HUMAN	0.4	0.038	0.49	0.04
Mitochondrial import receptor subunit TOM22 homolog	TOM22_HUMAN	0.6	0.12	0.48	0.15
Vesicle-associated membrane protein 2	VAMP2_HUMAN	1.5	0.036	2.11	0.029
Vesicle-associated membrane protein-associated protein B/C	VAPB_HUMAN	1.5	0.27	1.25	0.25

A number of proteins were analyzed by Western blot and quantified for comparison to the fold change values determined by MS/MS LFQ. The fold changes and p-values calculated by both methods gave comparable results, providing confidence in the LFQ quantification method used in this study. The data provided here refer to AF22 cells challenged with oAβ relative to untreated AF22 cells. Fold changes determined by MS/MS are described in Table 1 and Supplementary Table S3. Western blots are provided in Supplementary Fig. S2. For MS/MS data: T-test with Benjamini-Hochberg correction, n = 14-16. For WB data: T-test, n = 6-7 per group.

### oAβ treatment elicits significant changes to the neuronal phosphoproteome

Given the importance of phosphorylation in ND-related proteins, we analyzed the phosphoproteome in differentiated human neurons. To achieve this, TiO_2_ enrichment was performed prior to nLC-MS/MS analysis and the resulting data was analyzed in a similar fashion to that of the proteome. In addition to phosphorylation, the dataset was searched for other PTMs (oxidation, methylation, acetylation), but none of these were found significantly changed in this sample. When searching the dataset for phosphorylation PTMs, we observed a significant alteration in 5 phosphorylated proteins: increases to the α-tubulin acetylating protein isoform 2 of alpha-tubulin N-acetyltransferase 1 (fold change 9.9, p = 0.002), isoform 2 of protocadherin-1 (fold change 11, p = 0.014), isoform 3 of periphilin-1 (fold change 1.7, p = 0.022), eukaryotic translation initiation factor 4B (fold change 1.6, p = 0.031), and a decrease in phosphorylated RNA-binding protein with serine-rich domain 1 (fold change 0.4, p = 0.043) (Table 3). Similar to the experiments with the unenriched proteome, we have also provided a list of proteins of interest to NDs that were identified while searching for phosphorylation PTMs (Supplementary Table S4), or without any search filters (Supplementary Table S5). Of particular interest, was the increase in phosphorylated tau in response to oAβ addition (fold change 1.3, p = 0.10) but this effect did not reach statistical significance. Similarly, the AD-associated tau kinase Casein kinase Iε was also increased (fold change 4.1, p = 0.053).

**Table 3:** Changes in the AF22 phosphoproteome resulting from 24 h oAβ treatment.

Identified Proteins	Protein ID	T-Test (p-value)	Fold Change	PTM
Isoform 2 of Alpha-tubulin N-acetyltransferase 1	ATAT_HUMAN	0.002	9.9	Phosphorylation
Eukaryotic translation initiation factor 4B	IF4B_HUMAN	0.031	1.6	Phosphorylation
Isoform 2 of Protocadherin-1	PCDH1_HUMAN	0.014	11.0	Phosphorylation
Isoform 3 of Periphilin-1	PPHLN_HUMAN	0.022	1.7	Phosphorylation
Isoform 3 of RNA-binding protein with serine-rich domain 1	RNPS1_HUMAN	0.043	0.4	Phosphorylation

The provided table depicts proteins with significant changes in expression (p < 0.05, T-test with Benjamini-Hochberg correction) in the phosphoproteome of AF22 cells challenged with oAβ relative to untreated AF22 cells. The proteins detailed in this table were identified after enrichment with TiO_2_. n = 11 (control), n = 8 (oAβ treated).

### Phosphorylation of tau at Ser208 may be an early PTM elicited by oAβ

One of the most important, and therefore most studied, aspects of NDs is the variable phosphorylation of the tau protein, which influences its microtubule binding efficiency, but also misfolding and self-aggregation. We examined the phosphorylation of tau in our PPE samples (Table 4), and identified that serine 208 (defined from the longest tau isoform, 2N4R containing 441 amino acids) was differentially phosphorylated in response to oAβ at this early time point, where 75% of the treated samples displayed phosphorylation at Ser208 (n = 8), while only 27% of the control cells had phosphorylation at this residue (n = 11). Other commonly studied phosphorylation sites such as Thr181 (epitope of antibody AT270) Ser202/Ser205 (epitope of antibody AT8), Thr231 (epitope of antibody AT180), Ser262, Ser396/Ser404 (epitope of antibody PHF-1) did not display appreciable differences in their tau phosphorylation at this early time point. We suggest that phosphorylation of tau at Ser208 could be an early marker of pathology in response to oAβ and warrants further investigation.

**Table 4:** Phosphorylation at Serine 208 is differentially expressed after 24 h oAβ challenge and may be a useful residue in the detection of early oAβ-induced effects.

Tau P10636-8 AA 441	Control samples with phosphorylation detected at residue: (n = 11)	Percentage of Control with detected residue	oAβ challenged samples with phosphorylation detected at residue: (n = 8)	Percentage of oAβ challenged with detected residue
181	1	9.09090909	1	12.5
197	2	18.1818182	2	25
198	2	18.1818182	2	25
199	8	72.7272727	6	75
202	11	100	8	100
205	3	27.2727273	3	37.5
208	3	27.2727273	6	75
212	1	9.09090909	0	0
217	1	9.09090909	0	0
231	2	18.1818182	0	0
235	9	81.8181818	7	87.5
238	2	18.1818182	0	0
262	3	27.2727273	1	12.5
394	1	9.09090909	0	0
396	5	45.4545455	5	62.5
400	9	81.8181818	8	100
403	2	18.1818182	0	0
404	11	100	8	100

Canonical phosphorylation sites such as 181, 202, 231, 262 and 404 do not show any appreciable changes at this early stage. The table depicts all detected phosphorylated tau residues for both AF22 cells challenged with oAβ and untreated AF22 cells. Phosphorylations detailed in this table were identified after TiO_2_ enrichment. Amino acid number corresponds to full-length 2N4R tau (441 residues; UniProt accession P10636-8), n = 11 (control) and n = 8 (oAβ).

### oAβ challenge elicits similar responses in V717I London APP mutant neurons as those observed in WT neurons

In addition to changes in the proteome observed in the WT APP neurons (AF22), we also examined the effects of oAβ stimulation on the proteome of iPSC-derived neurons from a donor harbouring the V717I London mutation (ADP2). We observed 11 significantly altered proteins (oAβ challenged ADP2 neurons relative to control ADP2 neurons; Table 5), many of which were related to the changes observed in the AF22 neurons (Table 1). In the ADP2 neurons, we observed the significant increase in proteasome subunit alpha type-4 (PSA4; fold change 4.3, p = 0.0084; Table 5), while its expression partner PSA3 was not significantly upregulated (fold change 4.0, p = 0.13; Table 1). In AF22 neurons, PSA3 that was upregulated (fold change 1.8, p = 0.038; Table 1), while PSA4 was not (fold change 1.0, p = 0.98; Table 5). Proteins belonging to the coatomer protein complex (COPI), which have been identified as risk factors for late-onset AD (LOAD), were differentially regulated in response to oAβ in both AF22 and ADP2 neurons. In ADP2, expression of Coatomer subunit gamma-1 (COPG1) was decreased in response to oAβ (fold change 0.2, p = 0.028; Table 5), while its expression partner Coatomer subunit delta (COPD) was unchanged (fold change 1.2, p = 0.88; Table 1). Conversely, in the AF22 neurons’ response to oAβ, COPG1 was not significantly altered (fold change 0.8, p = 0.66; Table 5), while COPD was significantly decreased (fold change 0.2, p = 0.026; Table 1). Similar to the experiments with the AF22 proteome, we searched the dataset for PTMs, however we only observed 2 proteins with significant changes in the ADP2 dataset: acetylated guanine nucleotide-binding protein G(I)/G(S)/G(T) subunit beta-1 was found to be increased in the oAβ stimulated group (fold change 4.2, p = 0.019; Supplementary Table S2), while phosphorylated DPYL2, which is associated with NFTs, was observed exclusively after oAβ challenge (fold change INF, p = 0.028; Supplementary Table S2). A curated list of ND relevant proteins that did not reach statistical significance is provided in Supplementary Table S3.

**Table 5:** Significantly up- and downregulated proteins in the proteome of ADP2 cells resulting from 24 h oAβ treatment.

Identified Proteins	Protein ID	AF22 T-Test (p-value)	AF22 Fold Change	ADP2 T-Test (p-value)	ADP2 Fold Change
Alpha-2-macroglobulin receptor-associated protein	AMRP_HUMAN	0.33	2.1	0.021	INF
Coatomer subunit gamma-1	COPG1_HUMAN	0.66	0.8	0.028	0.2
Isoform 2 of Serine/threonine-protein kinase DCLK2	DCLK2_HUMAN	0.33	0.5	0.031	8
Isoform Cytoplasmic of Fumarate hydratase, mitochondrial	FUMH_HUMAN	0.14	1.5	0.012	2.6
Cluster of Isoform 2 of Eukaryotic initiation factor 4A-II	IF4A2_HUMAN	0.93	1	0.029	0.5
Cluster of Isoform Delta 10 of Calcium/calmodulin-dependent protein kinase type II subunit delta	KCC2D_HUMAN	0.68	1.2	0.011	2.5
Isoform 2 of Nucleosome assembly protein 1-like 1	NP1L1_HUMAN	0.55	0.9	0.041	2
Proteasome subunit alpha type-4	PSA4_HUMAN	0.98	1	0.0084	4.3
60 S acidic ribosomal protein P1	RLA1_HUMAN	0.85	0.9	0.032	2
Isoform 2 of Neuronal-specific septin-3	SEPT3_HUMAN	0.29	1.4	0.037	3.8
Serine/arginine-rich splicing factor 2	SRSF2_HUMAN	0.76	0.9	0.043	2.3

The provided table depicts proteins with significant changes in expression (p < 0.05, T-test with Benjamini-Hochberg correction) in cells challenged with oAβ relative to untreated cells (i.e. ADP2 + oAβ relative to ADP2 control). ADP2: n = 7 (control), n = 7 (oAβ treated). Data represent analysis of whole proteome preparations. AF22 columns provided for comparison. AF22: n = 16 (control), n = 14 (oAβ treated). ND = Not detected.

## Discussion

One of the most studied hallmarks of AD is the progressive propagation of Aβ pathology, thought to be caused by the spreading of oAβ among interconnected brain regions from neuron to neuron^5,22^, further reviewed in^1–4^. In this work, we aim to elucidate intracellular protein changes elicited by oAβ upon naïve human neurons, thereby furthering our understanding of the early mediators involved in Aβ-induced pathogenesis. To do this, we used well characterized iPSC-derived ltNES cells, which upon differentiation, are electrophysiologically mature and display mature neuronal markers^14–21^. Since Aβ oligomers are thought to be the most relevant aggregates in AD pathology, we challenged the differentiated neurons with 1 µM oAβ over 24 h to mimic the early phase of oAβ challenge, similar to previous studies^22,23,26–30^. Previous studies have shown that concentrations of up to 3 µM oAβ 1-42 exist within neurons of the AD brain^31^, while the concentrations of oAβ used in this study may reflect those in the immediate vicinity of amyloid plaque cores, the so-called halo region^32,33^. Using nLC-MS/MS and LFQ, we investigated the changes to the proteome and phosphoproteome elicited by this oAβ challenge. Using this unbiased approach, we were able to identify a number of proteins of interest that support previous findings^34,35^, but also proteins that extend our current understanding of early amyloid pathology.

Analysis of the AF22 proteome in response to oAβ revealed 42 significantly up- and downregulated proteins. These included a significant reduction to the mRNA processing protein Heterogeneous nuclear ribonucleoprotein F (hnRNP-F). The hnRNP family of proteins are RNA binding proteins that perform many functions, including nucleic acid metabolism, translational regulation and alternative splicing (reviewed in^36^). In AD, ALS and FTLD, there is dysregulation of hnRNP expression (especially A/B isoforms, but also H/F), which are thought to exacerbate memory impairments^37–41^. Conversely, we observed increases in the Aβ-binding protein SGTA^42^ and neuronal growth-related protein DPYL5. The DPYL family of proteins, also known as Collapsin response mediator proteins (CRMPs) are neuronally expressed proteins important to axon and neurite formation and have been linked to neurodegeneration in AD, PD and traumatic brain injury^43,44^. In a recent study, it was shown that increased DPYL5 exacerbated memory loss in an AD mouse model^45^. A previous study also identified the expression of DPYL proteins to be significantly altered in response to oAβ 1-42, although this study had the limitations of being performed in neuroblastoma SH-SY5Y cells with an extremely large dose of oAβ (15 µM)^35^. Nevertheless, in their study Földi *et al*. found significant changes in proteins related to those observed in this work, including reductions in hnRNP-K, but contrary to our findings, a decrease in DPYL proteins. Previous studies have strongly suggested that Aβ and the ND-related, mRNA processing protein TDP-43 are related in the pathology of neurodegeneration. Animal studies have shown that chronic exposure of Aβ in the brain leads to TDP-43 pathology^46^ and that TDP-43 can induce the cross-seeding of Aβ into oAβ^47^. In human AD patients, more than half also display TDP-43 comorbidity^48–50^. Supporting the link between them, we found a significant reduction in the expression of TDP-43 after challenge with oAβ in the AF22 neurons, but not ADP2. We speculate that the reduction in TDP-43 expression that we observed in response to oAβ after 24 h may reflect the neuron’s attempt to limit the damage caused by TDP-43 to cells during acute oAβ exposure. In animal models of AD, TDP-43 levels are initially indistinguishable from the WT but increase over time during chronic oAβ exposure. Given our observations, we suggest that there is an initial reduction in TDP-43 expression during initial oAβ exposure, which gradually increases over time as the cell is exposed to Aβ chronically^51,52^. This may also be a contributing factor as to why TDP-43 reduction was not as robust in the ADP2 neurons, owing to their chronic exposure to endogenously generated Aβ 1-42 during differentiation^53^. Altogether, this model points toward widespread RNA processing dysregulation in the early stages of oAβ challenge^54^.

Given that hyperphosphorylation is important to the neurodegenerative process in multiple ND’s, we also investigated the phosphoproteome in AF22 neurons after 24 h incubation with oAβ, with a specific interest in the differential phosphorylation of tau. Following phosphoenrichment with TiO_2_, we found that tau expression was elevated after exposure to oAβ, but that this elevation did not achieve statistical significance (fold change 1.3, p = 0.10). Furthermore, we identified the differential phosphorylation of tau at serine 208. At Ser208, we found that 75% of the oAβ-exposed samples displayed phosphorylation, whereas only 27% of the control samples experienced phosphorylation at the same site. Other canonical tau phosphorylation sites, including early (Thr181, Ser202/Ser205 and Ser262), intermediate (Thr231 and Thr212/Ser214) and late residues (Ser396/Ser404 and Ser422)^6,55,56^ were not remarkably altered by exposure to oAβ for 24 h. In future studies, it will be imperative to confirm this finding using other methods (e.g. Western blot), however there are currently no commercially available antibodies specific to tau phosphorylation at Ser208 alone. Nevertheless, this finding indicates that phosphorylation of tau at Ser208 may be an early consequence of oAβ exposure. Following independent verification of this finding, this tau phosphoepitope should be further investigated as a potential early marker of tauopathy in Aβ related NDs. In addition to tau itself, we observed an increase (p = 0.053) in CK1ε, a member of the Casein Kinase 1 family (CK1 isoforms include α1, α2, γ1, γ2, γ3, δ, and ε; family also includes Tau-tubulin kinases (TTBK1 and TTBK2)), which are known to be major tau and TDP-43 kinases^46,57,58^, and phosphorylate tau at Ser208^59^. Furthermore, CK1ε specifically, has been shown to be significantly elevated in AD and contributes to increased expression of Aβ^7,58^. CK1ε also functions as a major regulator of circadian rhythms^60^, leading speculation that these disturbances to CK1ε expression in AD could be related to the sleep disturbances experienced by patients. Previous *in vivo* experiments have shown that treatment of 5xFAD mice with recombinant nuclease-sensitive element-binding protein 1 (YBOX 1) results in decreased levels of Aβ in the brain, but also inhibits the fibrillization of Aβ 1-42 *in vitro*^61^. We show that oAβ exposure caused a decrease in phosphorylated YBOX 1 in the exposed neurons (fold change 0.3, p = 0.054). The effect of phosphorylation on YBOX 1 in relation to AD is unknown, but YBOX 1 phosphorylation is thought to enhance the activation of transcription of NF-κB during colon cancer^62,63^.

The APP London (V717I) mutation is one of the most common familial APP mutations of AD, with patients experiencing the onset of clinical dementia at an average age of 59^64^. This mutation results in an increased Aβ42:Aβ40 ratio in the brain and in patient derived iPSCs through increased production of Aβ 1-42^53,65–67^. oAβ challenge of differentiated ltNES neurons harbouring this mutation (ADP2) resulted in fewer significant changes to the proteome relative to the WT APP (AF22) neurons (11 vs. 42, respectively). It is possible that the additional exposure to 1 µM oAβ did not elicit a robust response, considering the elevated baseline expression of Aβ 1-42 in V717I mutants. Using LFQ, we identified that total Aβ was increased in the oAβ exposed ADP2 cells relative to the control ADP2 cells, however the difference did not achieve statistical significance, presumably relating to the baseline expression of Aβ 1-42 in the ADP2 neurons^53^. It is worth noting that during differentiation, ADP2 neurons experience a much greater degree of spontaneous cell death compared to AF22, likely a result of the increased expression of endogenous Aβ 1-42. Since whole proteome analysis requires small amounts of protein input, this did not prohibit proteomic analysis, however phosphoenrichment requires the input of large quantities of peptides (≈1000 µg), making analysis of the ADP2 phosphoproteome unattainable. Nevertheless, we identified a number of proteins that were upregulated following oAβ exposure, including the increased expression of the tricarboxylic acid cycle (TCA cycle)-related protein Fumarate hydratase, mitochondrial (FUMH), which was also found to be upregulated in response to oAβ by Földi *et al*.^35^. Additionally, in the whole proteome analysis of ADP2, we found that DPYL2 (AKA CRMP2), a member of the aforementioned AD-associated DPYL protein family, was found to be phosphorylated, and this phosphorylated DPYL2 was only observed after the oAβ challenge. DPYL2 phosphorylation is regulated by the neurodegenerative disease related kinase GSK-3β, with the phosphorylated form being an important mediator of axon elongation and repair^43,44^. Interestingly, phosphorylated DPYL2 was identified as an antigen of the 3F4 monoclonal antibody, which was raised against partially purified PHFs and NFTs, indicating that phosphorylated DPYL2 may be implicated in the formation of PHFs and NFTs in AD^68^. After oAβ exposure, we observed a statistically significant increase in PSA4 in the ADP2 neurons, while in AF22 neurons, its expression partner PSA3 was significantly increased. The oAβ-induced PSA protein increases observed in this work are further supported by a previous proteomics study that found these 20 s proteasomal proteins were differentially regulated in AD brains^69^. As the 20 S proteasome is important for the degradation of ubiquitinated and misfolded proteins^70^, its disruption at early stages of oAβ challenge, like those shown here, should be further investigated for their possible contributions to AD pathogenesis.

In both ADP2 and AF22 neurons, exposure of oAβ caused a significant downregulation of coatomer complex proteins (COPG1 in ADP2 and COPD in AF22). Both COPD and COPG1 are subunits of the larger protein complex COPI. COPI-coated vesicles are responsible for the retrograde transport of proteins from the Golgi to the endoplasmic reticulum (ER, reviewed in^71^). COPI function is important to AD pathogenesis, as it regulates APP trafficking, maturation and therefore production of Aβ peptides^72^. Furthermore, SNPs in the COPI subunits gamma-1 and delta have been identified as risk factors for the development of LOAD^73^. Upon COPI depletion, the LOAD related protein TREM2 fails to mature, impairing its cell-surface expression, instead accumulating in the ER–Golgi intermediate compartment^74^. Additionally, mice with a missense mutation in COPD develop protein accumulations, ER stress, and neurofibrillary tangles^75^. COPI downregulation leads to excessive accumulation of APP at the Golgi, thereby hindering its maturation into Aβ and cell surface expression, culminating in less Aβ load, reduced Aβ plaque deposition and improved memory^72,73^.

As recently shown by Volpato *et al*., it is important to consider the reproducibility of iPSC-derived cells with respect to expressional variations between laboratories^76^. In this study, we have utilized thoroughly characterized iPSC-derived ltNES cells, which have been widely used in several different laboratories to derive neurons for *in vitro* studies^14–21^. Importantly, these cells have been demonstrated to maintain stable morphology, differentiation potential and gene expression profiles over more than 100 passages^14^ and display consistent expression profiles across different laboratories^15,16,18,19^. Using the same differentiation scheme as previously described for these ltNES cells^14^, we found similar proportions of neurons to glia upon differentiation as those previously described^14,16,18,19^. While each of these ltNES cells have been shown to retain consistent morphology and gene expression they are each derived from different individual donors, with individual genotype and epigenetic modifications resulting in differences in baseline mRNA and protein levels. This could result both in differences in expression, resilience to oAβ and morphological differences, e.g. levels of RNA expression can affect nuclear size^77^. In this study we have examined the effects of the oAβ challenge upon each ltNES cell-type separately (i.e. AF22 + oAβ was compared to AF22 control) to avoid the confounding factor of different baselines. Further studies including additional genotypes and other models will be important to confirm the results in this study. In addition, it will be interesting to study the effect of other misfolded protein aggregates, which could activate similar but also additional pathways. Despite these potential future expansions, we believe that the results obtained here give important indications of the early oAβ induced changes.

In this study, we have demonstrated that iPSC-derived ltNES neurons are a useful model for the study of oAβ-induced changes to the proteome and phosphoproteome. Using nLC-MS/MS and subsequent LFQ, we identified a number of proteins that were differentially regulated, some of which have been associated with neurodegenerative disorders including AD, but also several that are previously unknown to be involved in these diseases. In this way, we have shown that TDP-43, hnRNPs, DPYLs and COPI proteins are among the earliest proteins affected by the uptake of oAβ in neurons. Additionally, oAβ induced the phosphorylation of tau at serine 208, which should be further investigated as a potential early marker of tau pathology. Together, these findings indicate that there are widespread alterations to the neuronal proteome within 24 h of oAβ uptake and provides new targets for the further study of early mediators of AD pathogenesis.

## Materials and Methods

### Cell culture

Well described iPSC-derived neuronal progenitor cells, long term neuroepithelial-like stem cells (ltNES), lineages AF22 (WT) and ADP2 (V717I London APP mutant) were kindly provided by Anna Falk (Karolinska Institute, Sweden)^14–21^. iPSC-derived cells were expanded in DMEM/F12 with Glutamax (Gibco), supplemented with EGF (10 ng/mL; Peprotech) and FGF2 (10 ng/mL; Peprotech), 1% N2 (Gibco), 0.1% B27 (Gibco), 1% Pen/Strep (Lonza). Once confluent, cells were differentiated for 60 days to obtain neuronal phenotypes^14^, followed by the addition of 1 µM amyloid beta oligomers (oAβ) for 24 h. Next cells were thoroughly washed, trypsinized and lysed in 4% SDS lysis buffer supplemented with protease inhibitor (HALT protease inhibitor cocktail; Thermo Scientific) and phosphatase inhibitor (PhosSTOP; Roche). Cell lysates were stored at −80 °C until use. Cell differentiation media was as follows: 1:1 mixture of Neurobasal and DMEM/F12 with Glutamax, supplemented with 1.5% N2, 1% B27 (all from Gibco) and 1% Pen/Strep (Lonza), with ½ media changed every day. These cells have been demonstrated to maintain stable morphology, differentiation potential and gene expression profiles over more than 100 passages^14^, however the cells used in this study did not exceed passage 40.

### Oligomerization of Aβ 1-42

Aβ oligomers were generated and confirmed by size exclusion chromatography (SEC) as previously described^22,23,27,78,79^. Recombinant Aβ 1-42 peptides (Innovagen) were dissolved in 1,1,1,3,3,3-hexafuoro-2-propanol (HFIP, Sigma-Aldrich) and vacuum dried overnight. Aβ 1-42 was first re-suspended in 44 µl DMSO (Sigma-Aldrich) and then diluted to a final concentration of 100 µM in HEPES 20 mM pH 7.4, vortexed, sonicated for 10 min and incubated at 4 °C overnight. After the overnight incubation, the sample was vacuum dried, re-suspended in 500 µl NH_4_HCO_3_ 50 mM pH 8.5. The oligomeric Aβ 1-42 was separated from the monomeric form with SEC. A Sephadex 75 10/300 GL column coupled to a liquid chromatography system (ÄKTA pure, GE Healthcare) was equilibrated with NH_4_HCO_3_ 50 mM pH 8.5 and 500 µl of sample was injected into the column. To estimate the molecular weight of the Aβ species, LMW gel filtration calibration kits (GE Healthcare) were used. Oligomeric and monomeric Aβ species were eluted at a flow rate of 0.5 ml/min, collected and lyophilized. Then, Aβ species were re-suspended in PBS and quantified spectrophotometrically at 215 nm by using the Aβ 1-42 extinction coefficient (Aβ 1-42 ɛ214 nm = 75,887 M-1 cm-1) according to Lambert–Beer’s law. Protein aliquots were stored at −80 °C. A representative SEC chromatogram of the Aβ oligomer preparations is provided in Supplementary Fig. S1.

### Protein extraction and digestion

Samples were lysed in SDS buffer (4% SDS, 25 mM HEPES pH 7.6, 1 mM DTT in H_2_O supplemented with protease inhibitor (HALT; Thermo Scientific) and phosphatase inhibitor (PhosStop; Roche)) which sufficiently solubilized all proteins. Solubilized proteins were precipitated with acetone, air dried, and re-suspended in urea solution (8 M urea, 2 M thiourea, 25 mM ammonium bicarbonate (ABC), incubated for 30 min), followed by reduction and alkylation (25 mM DTT (15 min) and 75 mM iodoacetamide (15 min), respectively). To remove residual SDS, solutions were washed five times with urea wash solution (8 M urea, 25 mM ABC) with 3 kDa Amicon spin filters (Merck Millipore), followed by removal of urea by washing the solution five times with 25 mM ABC. At this stage, the protein in solution (25 mM ABC) was digested in trypsin (Sigma-Aldrich; 1:25 w/w trypsin:protein) for 2 h at 37 °C and repeated overnight at 37 °C, then dried in a speed vacuum concentrator. To ensure complete removal of contaminants, peptides were desalted with C18 spin columns according to manufacturer’s instructions (Pierce), dried and stored at −80 °C until use. Peptides were reconstituted in 0.1% formic acid in MilliQ H_2_O, and approximately 0.25 µg was used in LC-MS/MS analysis.

For analysis of the phosphoproteome, proteins were extracted and digested as described above, but subjected to phosphoenrichment (PPE) with TiO_2_ spin columns (Pierce) after trypsinization instead of C18 cleanup. PPE was performed according to manufacturer’s instructions.

### LC-MS/MS analysis

Peptides were separated by reverse phase chromatography on a 20 mm × 100 μm C18 pre-column followed by a 100 mm × 75 μm C18 column with particle size 5 μm (NanoSeparations) at a flow rate of 300 nL/min. Samples were loaded using an EASY-nLC II (Thermo) by linear gradient of 0.1% formic acid in water (A) and 0.1% formic acid in acetonitrile (B) (0–100% B in 90 min for proteome analysis, 0–100% B in 120 min for phosphoproteome analysis). Automated online analyses were performed with an LTQ Orbitrap Velos Pro hybrid mass spectrometer (Thermo) with a nano-electrospray source.

### Database searches and label-free quantification

Database searches and label-free quantification (LFQ) were performed as described previously^80–82^. Raw files were searched using Sequest HT in Proteome Discoverer (Thermo; version 1.4.0.288) and X! Tandem (The GPM, thegpm.org; version CYCLONE (2010.12.01.1)). Sequest was searched against a UniProt Human database (available at UniProtKB website: https://www.uniprot.org/taxonomy/9606) assuming the digestion enzyme trypsin. X! Tandem was set up to search a reverse concatenated subset of the Swiss-Prot database. Sequest and X! Tandem were searched with a fragment ion mass tolerance of 0.60 Da and a parent ion tolerance of 10.0 PPM. Carbamidomethyl of cysteine was specified in Sequest and X! Tandem as a fixed modification. Methyl of aspartic acid, glutamic acid, oxidation of methionine, acetyl of lysine and phospho of serine, threonine and tyrosine were specified in Sequest and X! Tandem as variable modifications.

Scaffold (version Scaffold_4.8.7, Proteome Software Inc., Portland, OR) was used to validate MS/MS based peptide and protein identifications. Identifications were based on a minimum of 1 unique peptide, 99% protein identification probability and 95% peptide identification probability (using the Scaffold Local FDR algorithm). Protein probabilities were assigned by the Protein Prophet algorithm^83^, resulting in decoy FDRs: 3.6% AF22 phosphoproteome, 3% AF22 proteome and 1.4% ADP2 proteome. Proteins that contained similar peptides and could not be differentiated based on MS/MS analysis alone were grouped to satisfy the principles of parsimony. Proteins sharing significant peptide evidence were grouped into clusters.

LFQ analysis of peptides was performed using the sum of the intensities of the peaks in the MS/MS spectra (Normalized Total TIC; normalized to reduce run-to-run variations), and quantitative differences were statistically analyzed by a T-Test with Benjamini-Hochberg correction. Differences with p-values <0.05 were considered statistically significant.

### Western blotting

To verify the validity of the quantification obtained by LC-MS/MS and Scaffold LFQ analysis, we analyzed protein expression in AF22 neurons by Western blotting with several antibodies. Protein concentrations were determined with DC Assay (Bio-Rad), and approximately 15 µg protein was loaded per lane on 4–20% gradient gels (CBS Scientific). After transfer to Nitrocellulose membranes (iBlot; Thermo Scientific), blots were incubated with primary antibodies overnight at 4 °C in TBST, followed by HRP-conjugated secondary antibodies for 90 minutes at ambient temperature in TBST. Membranes were visualized using Clarity ECL on a ChemiDoc MP imaging system (both Bio-Rad), followed by quantification with ImageJ. Membranes were stripped with Restore PLUS Western Blot Stripping Buffer (Thermo Scientific) for 10 minutes at ambient temperature. A list of antibodies used in this study and their dilutions can be found in Supplementary Table S1.

### Immunocytochemistry

In order to confirm the that our differentiation of AF22 and ADP2 cells resulted in predominantly neuronal populations, we analyzed the differentiated cells for the expression of neuron-specific markers NeuN and β-III Tubulin (Tuj1). Cells were fixed with 4% PFA for 10-20 minutes and washed thoroughly with PBS prior to permeabilization with 0.1% saponin and 5% FBS (in PBS) for 20 min. Cells were further permeabilized/blocked in a PBS buffer containing 0.02% Triton X-100, 0.1% BSA, and 0.05% Tween-20. Primary antibodies were incubated overnight at 4 °C followed by thorough washing and a 75-minute incubation at ambient temperature with secondary antibody. Cells were mounted using ProLong Gold Antifade Mountant with DAPI (Invitrogen). Antibody dilutions are presented in Supplementary Table S1. To quantify the proportion of neurons, AF22 (150 cells) and ADP2 (130 cells) cells were analyzed for DAPI (all nucleated cells) and NeuN-positivity (neurons). The proportions of NeuN-positive cells were compared between AF22 and ADP2 with an unpaired T-test.

## Supplementary information

Supplementary information.

## Data Availability

All relevant data analysed during this study are included in this published article (and its Supplementary Information files). The raw proteomics data are available from the corresponding author on reasonable request.
