# Supplementary material for: Oligomeric amyloid-β induces early and widespread changes to the proteome in human iPSC-derived neurons
Source: Sci Rep. 2020 Apr 16;10:6538. doi: 10.1038/s41598-020-63398-6 (PMC7162932; doi:10.1038/s41598-020-63398-6)
Supplement: Supplementary file 1 — Supplementary information. [file 41598_2020_63398_MOESM1_ESM.docx]

**Oligomeric amyloid-β induces early and widespread changes to the proteome in human iPSC-derived neurons**

Christopher Sackmann, Martin Hallbeck

Supplementary Tables

Supplementary Table S1. List of antibodies used in the study.

| Antibody | Dilution | Distributor | Product Number |
| --- | --- | --- | --- |
| VAMP2 | 1:1000 | Cell Signaling Technology | 13508 (Clone D601A) |
| Rab7a | 1µg/mL | R&D Systems | AF7789 |
| VAPB | 1:1000 | R&D Systems | MAB58551 (Clone 736904) |
| TOMM22 | 0.4µg/mL | Novus Biologicals | NBP1-80671 |
| Calreticulin | 1:1000 | Novus Biologicals | NB600-101 |
| TDP-43 | 1:1000 | Aviva Systems Biology | OAAB10063 |
| β-Actin | 1:10000 | Sigma-Aldrich | A5441 (Clone AC-15) |
| Goat anti-mouse-HRP | 1:2000 | Dako | P0447 |
| Goat anti-rabbit-HRP | 1:2000 | Dako | P0448 |
| Rabbit anti-rat-HRP | 1:2000 | Dako | P0450 |
| GAPDH-HRP | 1:10000 | Novus Biologicals | NB300-328H (Clone 2D4A7) |
| β-III Tubulin | 1:500 | R&D Systems | MAB1195 (Clone Tuj1) |
| NeuN | 1:500 | Merck Millipore | MAB377 (Clone A60) |
| Goat anti-mouse Alexa Fluor-488 | 1:1000 | Invitrogen | A11029 |

Supplementary Table S2. Post-translational modifications detected in whole proteome preparations of AF22 and ADP2 cells induced by 24h oAβ treatment. The provided table depicts proteins with significant changes in expression (p<0.05, T-test with Benjamini-Hochberg correction) in AF22 and ADP2 cells challenged with oAβ relative to untreated AF22 and ADP2 cells (i.e. AF22+oAβ relative to AF22 control and ADP2+oAβ relative to ADP2 control). AF22: n=16 (control), n=14 (oAβ treated). ADP2: n=7 (control), n=7 (oAβ treated). ND = Not detected.

| Identified Proteins | Protein ID | AF22  T-Test (p-value) | AF22  Fold Change | ADP2  T-Test (p-value) | ADP2  Fold Change | PTM |
| --- | --- | --- | --- | --- | --- | --- |
| 14-3-3 protein epsilon | 1433E_HUMAN | 0.0097 | 0.3 | 0.13 | 2.5 | Acetylation |
| Cluster of Isoform 2 of Elongation factor 1-delta | EF1D_HUMAN | 0.035 | INF | 0.22 | 0.0 | Acetylation |
| Guanine nucleotide-binding protein G(I)/G(S)/G(T) subunit beta-1 | GBB1_HUMAN | 0.49 | 1.3 | 0.019 | 4.2 | Acetylation |
| Cluster of Isoform 3 of Histone-binding protein RBBP4 | RBBP4_HUMAN | 0.05 | 0.0 | 0.34 | 0.0 | Acetylation |
|  |  |  |  |  |  |  |
| Cluster of Tubulin beta-3 chain | TBB3_HUMAN | 0.036 | 0.07 | 0.74 | 0.7 | Methylation |
|  |  |  |  |  |  |  |
| Cluster of Alpha-internexin | AINX_HUMAN | 0.037 | 1.8 | ND | ND | Phosphorylation |
| Dihydropyrimidinase-related protein 2 | DPYL2_HUMAN | 0.96 | 1.0 | 0.028 | INF | Phosphorylation |
| Cluster of Isoform 3 of Heterogeneous nuclear ribonucleoprotein D0 | HNRPD_HUMAN | 0.048 | 0.06 | 0.95 | 0.9 | Phosphorylation |
| Cluster of Stathmin | STMN1_HUMAN | 0.046 | 2.1 | 0.061 | 4.7 | Phosphorylation |
|  |  |  |  |  |  |  |
| Cluster of Isoform Beta of Apoptosis regulator BAX | BAX_HUMAN | 0.006 | 0 | ND | ND | Oxidation |
| Emerin | EMD_HUMAN | 0.031 | INF | ND | ND | Oxidation |
| Ras-related protein Ral-A | RALA_HUMAN | 0.02 | 25 | ND | ND | Oxidation |

Supplementary Table S3. Selected proteins of interest demonstrating fold change in whole proteome preparations of AF22 and ADP2 cells induced by 24h oAβ treatment. The provided table depicts proteins with changes in expression (p<0.05, T-test with Benjamini-Hochberg correction) in AF22 and APD2 cells challenged with oAβ relative to untreated AF22 and ADP2 cells (i.e. AF22+oAβ relative to AF22 control and ADP2+oAβ relative to ADP2 control). AF22: n=16 (control), n=14 (oAβ treated). ADP2: n=7 (control), n=7 (oAβ treated). ND = Not detected.

| Identified Proteins | Protein ID | AF22  T-Test (p-value) | AF22  Fold Change | ADP2  T-Test (p-value) | ADP2  Fold Change |
| --- | --- | --- | --- | --- | --- |
| 14-3-3 protein beta/alpha | 1433B_HUMAN | 0.25 | 1.3 | 0.14 | 1.8 |
| 14-3-3 protein epsilon | 1433E_HUMAN | 0.78 | 1 | 0.3 | 1.6 |
| 14-3-3 protein eta | 1433F_HUMAN | 0.25 | 1.4 | 0.25 | 1.6 |
| 14-3-3 protein gamma | 1433G_HUMAN | 0.32 | 1.2 | 0.1 | 1.8 |
| 14-3-3 protein theta | 1433T_HUMAN | 0.44 | 1.2 | 0.28 | 1.4 |
| 14-3-3 protein zeta/delta | 1433Z_HUMAN | 0.37 | 1.1 | 0.3 | 1.4 |
| Serine/threonine-protein phosphatase 2A 65 kDa regulatory subunit A alpha isoform | 2AAA_HUMAN | 0.76 | 1.1 | 0.21 | 1.4 |
| Actin, cytoplasmic 1 | ACTB_HUMAN | 0.46 | 0.9 | 0.56 | 0.9 |
| Apolipoprotein E | APOE_HUMAN | 0.34 | 1.6 | 0.74 | 1.3 |
| Alpha-tubulin N-acetyltransferase 1 | ATAT_HUMAN | 0.36 | 0.7 | 0.75 | 1.3 |
| Calreticulin | CALR_HUMAN | 0.89 | 1 | 0.41 | 1.2 |
| Cyclin-dependent kinase 1 | CDK1_HUMAN | 0.44 | 0.4 | ND | ND |
| Isoform 3 of Drebrin | DREB_HUMAN | 0.62 | 1.1 | 0.49 | 1.4 |
| Flotillin-1 | FLOT1_HUMAN | 0.056 | 0.5 | 0.94 | 1 |
| Flotillin-2 | FLOT2_HUMAN | 0.9 | 0.9 | 0.29 | 5.2 |
| RNA-binding protein FUS | FUS_HUMAN | 0.17 | 1.3 | 0.39 | 1.6 |
| Glyceraldehyde-3-phosphate dehydrogenase | G3P_HUMAN | 0.27 | 0.8 | 0.79 | 1.1 |
| Glial fibrillary acidic protein | GFAP_HUMAN | 0.35 | 1.7 | 0.85 | 0.9 |
| Eukaryotic translation initiation factor 4B | IF4B_HUMAN | 0.39 | 2.3 | 0.12 | INF |
| Lysosome-associated membrane glycoprotein 1 | LAMP1_HUMAN | 0.22 | 0.5 | 0.89 | 0.9 |
| LAMP-2B of Lysosome-associated membrane glycoprotein 2 | LAMP2_HUMAN | 0.39 | 0.5 | 0.73 | 1.4 |
| Microtubule-associated protein 1B | MAP1B_HUMAN | 0.51 | 1.1 | 0.3 | 1.4 |
| Microtubule-associated protein 4 | MAP4_HUMAN | 0.3 | 1.4 | 0.15 | 1.9 |
| Microtubule-associated protein 2 | MTAP2_HUMAN | 0.054 | 1.5 | 0.2 | 2.1 |
| Serine/threonine-protein phosphatase PP1-beta catalytic subunit | PP1B_HUMAN | 0.43 | 0.8 | ND | ND |
| Serine/threonine-protein phosphatase PP1-gamma catalytic subunit | PP1G_HUMAN | 0.44 | 0.8 | 0.66 | 0.7 |
| Serine/threonine-protein phosphatase PP1-gamma catalytic subunit | PP1G_HUMAN | 0.66 | 0.9 | 0.66 | 0.7 |
| Serine/threonine-protein phosphatase 2A catalytic subunit beta isoform | PP2AB_HUMAN | 0.96 | 1 | 0.51 | 1.3 |
| Cluster of Isoform 2 of Serine/threonine-protein phosphatase 2B catalytic subunit alpha isoform | PP2BA_HUMAN | 0.58 | 1.3 | 0.64 | 1.5 |
| Serine/threonine-protein phosphatase 2B catalytic subunit beta isoform | PP2BB_HUMAN | 0.64 | 1.3 | 0.39 | 2.7 |
| Protein phosphatase 1G | PPM1G_HUMAN | 0.59 | 0.8 | 0.41 | 3.1 |
| Serine/threonine-protein phosphatase 5 | PPP5_HUMAN | 0.11 | 0.2 | ND | ND |
| Ras-related protein Rab-2A | RAB2A_HUMAN | 0.98 | 1 | 0.74 | 0.9 |
| Ras-related protein Rab-2B | RAB2B_HUMAN | 0.73 | 0.9 | 0.89 | 0.9 |
| Ras-related protein Rab-5A | RAB5A_HUMAN | 0.74 | 0.9 | ND | ND |
| Ras-related protein Rab-5B | RAB5B_HUMAN | 0.95 | 1 | 0.54 | 0.8 |
| Ras-related protein Rab-7a | RAB7A_HUMAN | 0.89 | 1 | 0.82 | 1.1 |
| RNA-binding protein with serine-rich domain 1 | RNPS1_HUMAN | 0.73 | 0.8 | ND | ND |
| SUMO-activating enzyme subunit 1 | SAE1_HUMAN | 0.41 | 0.7 | 0.43 | 4.1 |
| Isoform 2 of Sorting nexin-2 | SNX2_HUMAN | 0.64 | 1.3 | ND | ND |
| Sorting nexin-3 | SNX3_HUMAN | 0.34 | 1.5 | 0.5 | 1.7 |
| Sorting nexin-4 | SNX4_HUMAN | 0.47 | 0.4 | ND | ND |
| Cluster of Superoxide dismutase, mitochondrial | SODM_HUMAN | 0.64 | 1.2 | 0.44 | 1.9 |
| Signal transducer and activator of transcription 3 | STAT3_HUMAN | 0.42 | 0.2 | 0.62 | 1.9 |
| Small ubiquitin-related modifier 2 | SUMO2_HUMAN | 0.24 | 0.7 | 0.56 | 1.4 |
| Synaptic vesicle glycoprotein 2A | SV2A_HUMAN | 0.19 | 1.9 | 0.85 | 1.1 |
| Synapsin-1 | SYN1_HUMAN | 0.081 | 5.6 | 0.32 | 2.7 |
| Alpha-synuclein | SYUA_HUMAN | 0.4 | 1.9 | 0.53 | 2.3 |
| Beta-synuclein | SYUB_HUMAN | 0.47 | 1.9 | ND | ND |
| Gamma-synuclein | SYUG_HUMAN | 0.15 | 2.7 | 0.9 | 0.8 |
| Microtubule-associated protein tau | TAU_HUMAN | 0.25 | 1.8 | 0.33 | 1.8 |
| Tubulin beta-3 chain | TBB3_HUMAN | 0.7 | 1 | 0.48 | 1.1 |
| Mitochondrial import receptor subunit TOM22 homolog | TOM22_HUMAN | 0.12 | 0.6 | 0.18 | 0.4 |
| Mitochondrial import receptor subunit TOM40 homolog | TOM40_HUMAN | 0.45 | 0.7 | 0.59 | 1.5 |
| Mitochondrial import receptor subunit TOM70 | TOM70_HUMAN | 0.32 | 0.5 | 0.28 | 4.5 |
| Vesicle-associated membrane protein-associated protein A | VAPA_HUMAN | 0.34 | 1.7 | 0.71 | 0.8 |
| Vesicle-associated membrane protein-associated protein B/C | VAPB_HUMAN | 0.27 | 1.5 | 0.96 | 0.9 |
| Vacuolar protein sorting-associated protein 26A | VP26A_HUMAN | 0.23 | 0.2 | 0.69 | 1.9 |
| Vacuolar protein sorting-associated protein 26B | VP26B_HUMAN | 0.39 | 2 | 0.51 | 0.3 |
| Vacuolar protein sorting-associated protein 37B | VP37B_HUMAN | 0.74 | 1.2 | ND | ND |
| Vacuolar protein sorting-associated protein 29 | VPS29_HUMAN | 0.96 | 0.9 | ND | ND |
| Vacuolar protein sorting-associated protein 35 | VPS35_HUMAN | 0.95 | 1 | 0.72 | 1.2 |

Supplementary Table S4. Selected phosphorylated proteins of interest in the AF22 phosphoproteome resulting from 24h oAβ treatment. The data reflect analysis of the phosphoproteome following enrichment with TiO_2_, and depict protein expression changes (p<0.05, T-test with Benjamini-Hochberg correction) in AF22 cells challenged with oAβ relative to untreated AF22 cells. The peptide search criteria for the data presented in this table required detection of phosphorylation. n=11 (control), n=8 (oAβ treated).

| Identified Protein | Protein ID | T-Test (p-value) | Fold Change | PTM |
| --- | --- | --- | --- | --- |
| 14-3-3 protein gamma | 1433G_HUMAN | 0.880 | 1.2 | Phosphorylation |
| Isoform 2 of Ataxin-2-like protein | ATX2L_HUMAN | 0.089 | INF | Phosphorylation |
| Basic leucine zipper and W2 domain-containing protein 2 | BZW2_HUMAN | 0.410 | 0.0 | Phosphorylation |
| Drebrin | DREB_HUMAN | 0.220 | 0.3 | Phosphorylation |
| Excitatory amino acid transporter 1 | EAA1_HUMAN | 0.160 | 6.4 | Phosphorylation |
| Isoform 2 of Ephrin type-B receptor 2 | EPHB2_HUMAN | 0.088 | INF | Phosphorylation |
| Isoform 2 of Exocyst complex component 1 | EXOC1_HUMAN | 0.097 | INF | Phosphorylation |
| Glycogen synthase kinase-3 alpha | GSK3A_HUMAN | 0.870 | 1.2 | Phosphorylation |
| Glycogen synthase kinase-3 beta | GSK3B_HUMAN | 0.650 | 1.6 | Phosphorylation |
| Casein kinase I isoform epsilon | KC1E_HUMAN | 0.053 | 4.1 | Phosphorylation |
| Microtubule-associated protein 1A | MAP1A_HUMAN | 0.560 | 1.9 | Phosphorylation |
| Microtubule-associated protein 1B | MAP1B_HUMAN | 0.340 | 0.7 | Phosphorylation |
| Myristoylated alanine-rich C-kinase substrate | MARCS_HUMAN | 0.070 | 2.8 | Phosphorylation |
| Microtubule-associated protein 2 | MTAP2_HUMAN | 0.880 | 1.0 | Phosphorylation |
| Neurofilament medium polypeptide | NFM_HUMAN | 0.480 | 1.2 | Phosphorylation |
| Nuclear ubiquitous casein and cyclin-dependent kinase substrate 1 | NUCKS_HUMAN | 0.810 | 1.1 | Phosphorylation |
| Isoform 2 of Nuclear mitotic apparatus protein 1 | NUMA1_HUMAN | 0.078 | 0.1 | Phosphorylation |
| Raftlin | RFTN1_HUMAN | 0.070 | 1.7 | Phosphorylation |
| Reticulon-3 | RTN3_HUMAN | 0.410 | 0.0 | Phosphorylation |
| Reticulon-4 | RTN4_HUMAN | 0.056 | 0.1 | Phosphorylation |
| Small glutamine-rich tetratricopeptide repeat-containing protein alpha | SGTA_HUMAN | 0.066 | 1.9 | Phosphorylation |
| Serine/arginine repetitive matrix protein 2 | SRRM2_HUMAN | 0.082 | 0.5 | Phosphorylation |
| Synaptic vesicle glycoprotein 2A | SV2A_HUMAN | 0.410 | 0.0 | Phosphorylation |
| Microtubule-associated protein tau | TAU_HUMAN | 0.100 | 1.3 | Phosphorylation |
| Vesicle-associated membrane protein 4 | VAMP4_HUMAN | 0.210 | 0.0 | Phosphorylation |
| Vacuolar protein sorting-associated protein 4A | VPS4A_HUMAN | 0.750 | 0.8 | Phosphorylation |
| Nuclease-sensitive element-binding protein 1 | YBOX1_HUMAN | 0.054 | 0.3 | Phosphorylation |

Supplementary Table S5. Selected proteins of interest demonstrating fold change in the AF22 proteome resulting from 24h oAβ treatment. The data reflect analysis of the proteome following enrichment with TiO_2_, and depict protein expression changes (p<0.05, T-test with Benjamini-Hochberg correction) in AF22 cells challenged with oAβ relative to untreated AF22 cells. The peptide search criteria for the data presented in this table did not include a PTM filter. n=11 (control), n=8 (oAβ treated).

| Identified Proteins | Protein ID | T-Test (p-value) | Fold Change |
| --- | --- | --- | --- |
| 14-3-3 protein beta/alpha | 1433B_HUMAN | 0.260 | 1.7 |
| 14-3-3 protein epsilon | 1433E_HUMAN | 0.890 | 0.9 |
| 14-3-3 protein gamma | 1433G_HUMAN | 0.099 | 2.4 |
| 14-3-3 protein theta | 1433T_HUMAN | 0.250 | 2.1 |
| 14-3-3 protein zeta/delta | 1433Z_HUMAN | 0.740 | 1.1 |
| Alpha-tubulin N-acetyltransferase 1 | ATAT_HUMAN | 0.003 | 9.9 |
| Ataxin-2-like protein | ATX2L_HUMAN | 0.089 | INF |
| Serine/threonine-protein kinase DCLK2 | DCLK2_HUMAN | 0.079 | 0.0 |
| Excitatory amino acid transporter 1 | EAA1_HUMAN | 0.150 | 6.3 |
| Ephrin type-B receptor 2 | EPHB2_HUMAN | 0.090 | INF |
| Exocyst complex component 1 | EXOC1_HUMAN | 0.098 | INF |
| XLas-2 of Guanine nucleotide-binding protein G(s) subunit alpha isoforms XLas | GNAS1_HUMAN | 0.077 | 2.0 |
| Glycogen synthase kinase-3 alpha | GSK3A_HUMAN | 0.930 | 1.1 |
| Glycogen synthase kinase-3 beta | GSK3B_HUMAN | 0.640 | 1.6 |
| High mobility group protein B1 | HMGB1_HUMAN | 0.048 | 0.1 |
| Hematological and neurological expressed 1 protein | HN1_HUMAN | 0.097 | 0.2 |
| Heat shock protein HSP 90-alpha | HS90A_HUMAN | 0.064 | 0.5 |
| Eukaryotic translation initiation factor 4B | IF4B_HUMAN | 0.037 | 1.6 |
| Casein kinase I isoform epsilon | KC1E_HUMAN | 0.056 | 4.0 |
| Kinesin-like protein KIF21A | KI21A_HUMAN | 0.100 | 0.0 |
| Microtubule-associated protein 1B | MAP1B_HUMAN | 0.300 | 0.7 |
| Myristoylated alanine-rich C-kinase substrate | MARCS_HUMAN | 0.070 | 1.7 |
| Microtubule-associated protein 2 | MTAP2_HUMAN | 0.970 | 1.0 |
| Neurofilament medium polypeptide | NFM_HUMAN | 0.500 | 1.2 |
| Nuclear ubiquitous casein and cyclin-dependent kinase substrate 1 | NUCKS_HUMAN | 0.820 | 1.1 |
| Nuclear mitotic apparatus protein 1 | NUMA1_HUMAN | 0.084 | 0.1 |
| Protocadherin-1 (Isoform 2) | PCDH1_HUMAN | 0.014 | 11.0 |
| Phospholipid phosphatase-related protein type 4 | PLPR4_HUMAN | 0.089 | INF |
| Periphilin-1 | PPHLN_HUMAN | 0.023 | 1.7 |
| Ras-related protein Rab-12 | RAB12_HUMAN | 0.097 | INF |
| Raftlin | RFTN1_HUMAN | 0.068 | 2.9 |
| RNA-binding protein with serine-rich domain 1 | RNPS1_HUMAN | 0.045 | 0.4 |
| Reticulon-4 | RTN4_HUMAN | 0.060 | 0.1 |
| Small glutamine-rich tetratricopeptide repeat-containing protein alpha | SGTA_HUMAN | 0.071 | 1.9 |
| SLIT-ROBO Rho GTPase-activating protein 3 | SRGP3_HUMAN | 0.099 | 1.8 |
| Serine/arginine repetitive matrix protein 2 | SRRM2_HUMAN | 0.083 | 0.5 |
| Synaptic vesicle glycoprotein 2A | SV2A_HUMAN | 0.410 | 0.0 |
| Microtubule-associated protein tau | TAU_HUMAN | 0.240 | 1.2 |
| Cluster of Tubulin beta chain | TBB5_HUMAN | 0.180 | 0.1 |
| Vesicle-associated membrane protein 4 | VAMP4_HUMAN | 0.190 | 0.0 |
| Vacuolar protein sorting-associated protein 4A | VPS4A_HUMAN | 0.790 | 0.8 |
| NEDD4-like E3 ubiquitin-protein ligase WWP2 | WWP2_HUMAN | 0.076 | 7.1 |
| Nuclease-sensitive element-binding protein 1 | YBOX1_HUMAN | 0.053 | 0.2 |

Supplementary Figures
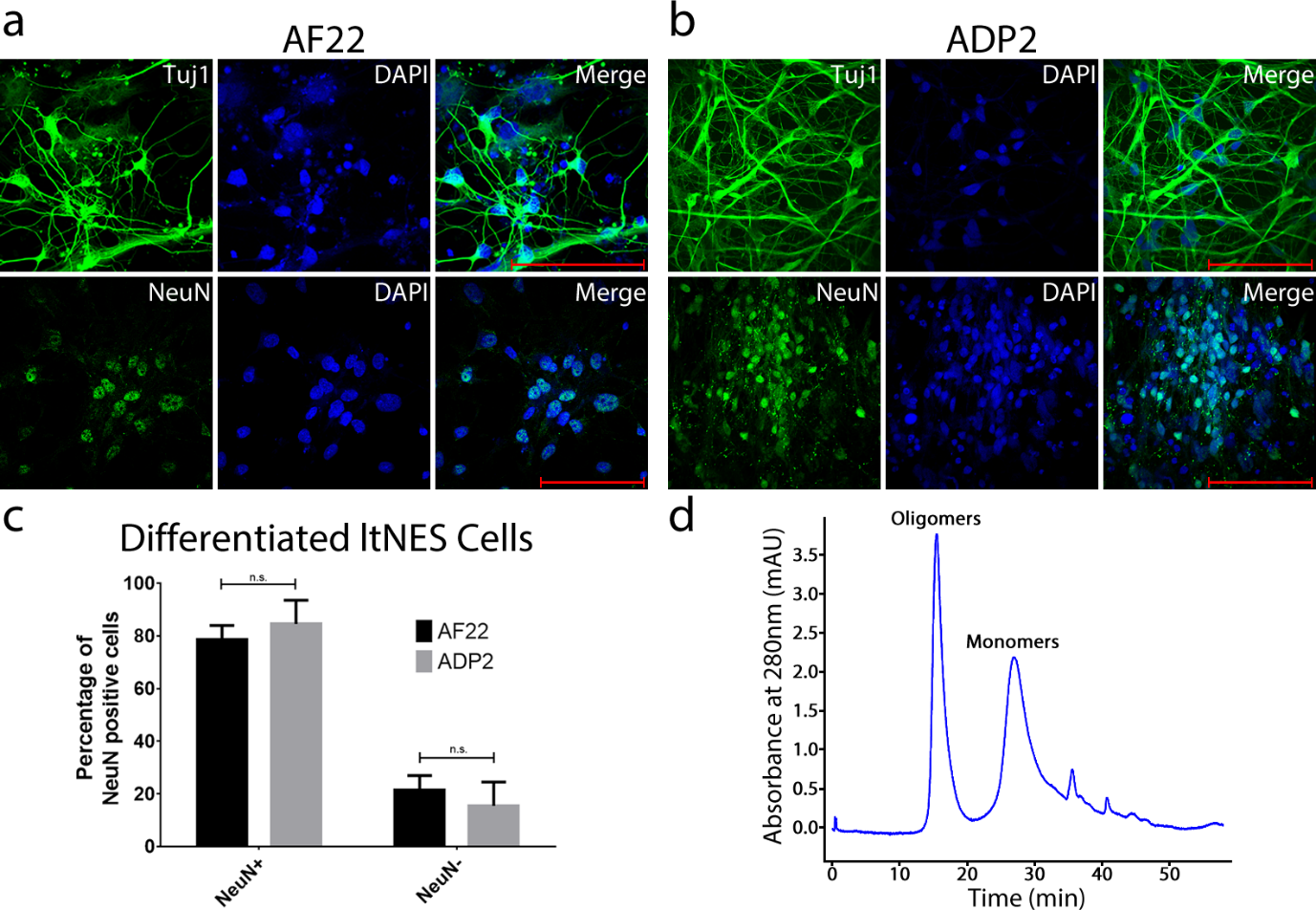


Supplementary Figure S1. Characterization of differentiated AF22 and ADP2 cells, and analysis of oAβ preparations by SEC. Widespread expression of the neuron-specific markers NeuN (neuronal nuclei) and β-III tubulin (Tuj1) indicates the neuronal nature of AF22 and ADP2 cells following differentiation (a, b). Expression of the neuronal nuclear marker NeuN was quantified in AF22 and ADP2 cells after differentiation, indicating that 78.5% (±5.4%) of AF22 cells were NeuN-positive while 84.5% (±9.0%) of ADP2 displayed NeuN-positivity (c). No statistically significant differences (n.s.) were observed in the proportion of NeuN-positive cells between AF22 and ADP2 cells (Unpaired T-test, n=150 cells (AF22), n=130 cells (ADP2)). Scale bar = 100µm. A representative SEC chromatogram indicates the presence of Aβ oligomers resulting from the aggregation process (d). Oligomeric and monomeric fractions eluted at retention times consistent to those previously reported using this fibrillization protocol (see Materials and Methods). For the confirmation of oligomeric Aβ content in the preparations shown here, absorbance was measured at 280nm (aromatic amino acids).


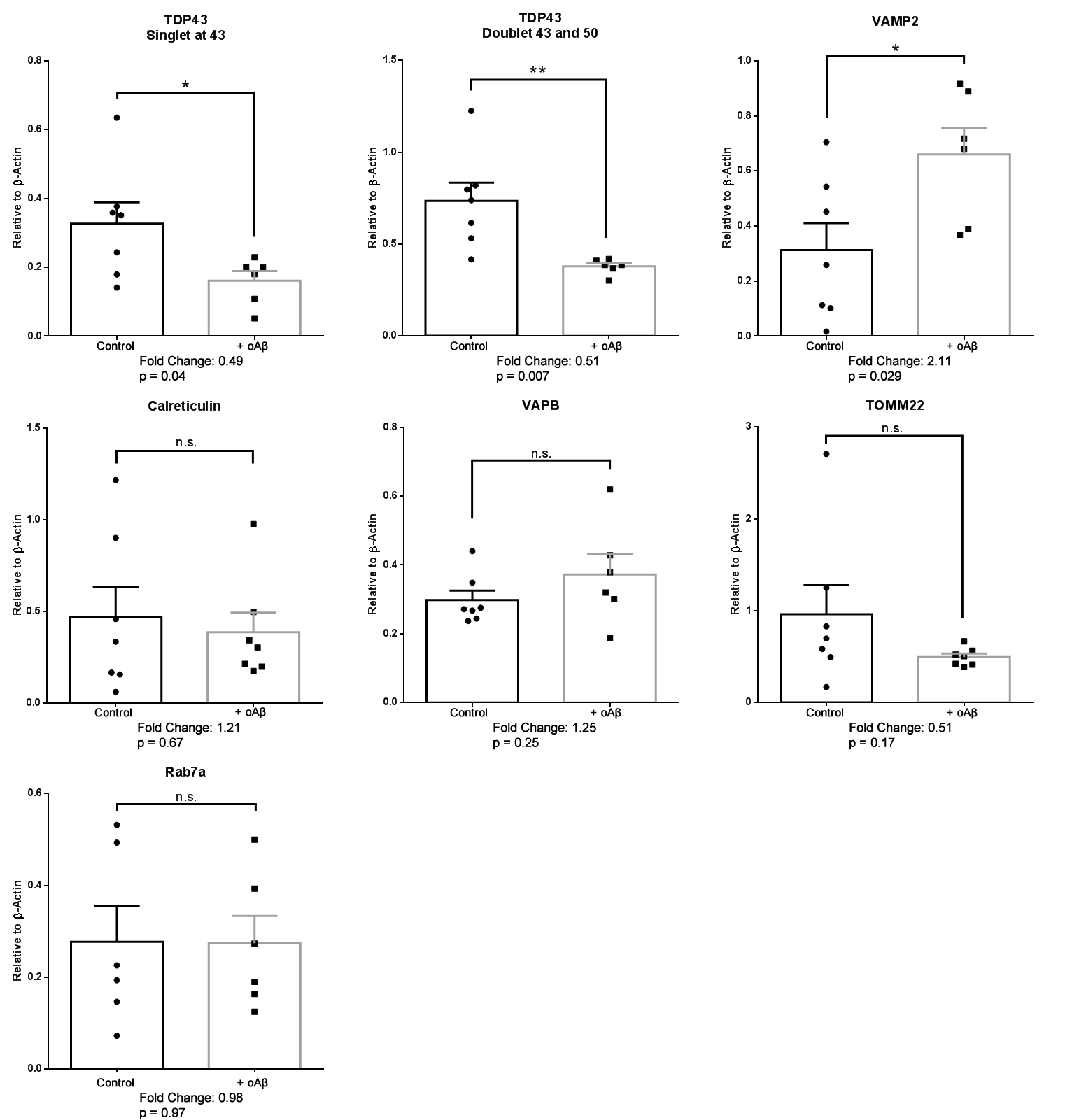
 Supplementary Figure S2. Protein quantification by Western blotting indicates a reduction in TDP-43 and increase in VAMP2 as early effects of oAβ treatment. Expression of TDP-43 (reduction), VAMP2 (increased) are significantly altered in response to oAβ, while VAPB, TOMM22, Rab7a and Calreticulin do not exhibit statistically significant changes. The fold changes measured by Western blot are comparable to those detected by LFQ in nLC-MS/MS (see Table 2). Fold change values represent AF22 cells challenged with oAβ relative to untreated AF22 cells. Representative Western blots corresponding to these data are presented in Supplementary Figure S3. Data are presented as mean ± SEM, *=p<0.05, **=p<0.01, as determined by T-test, n=6-7 per group.


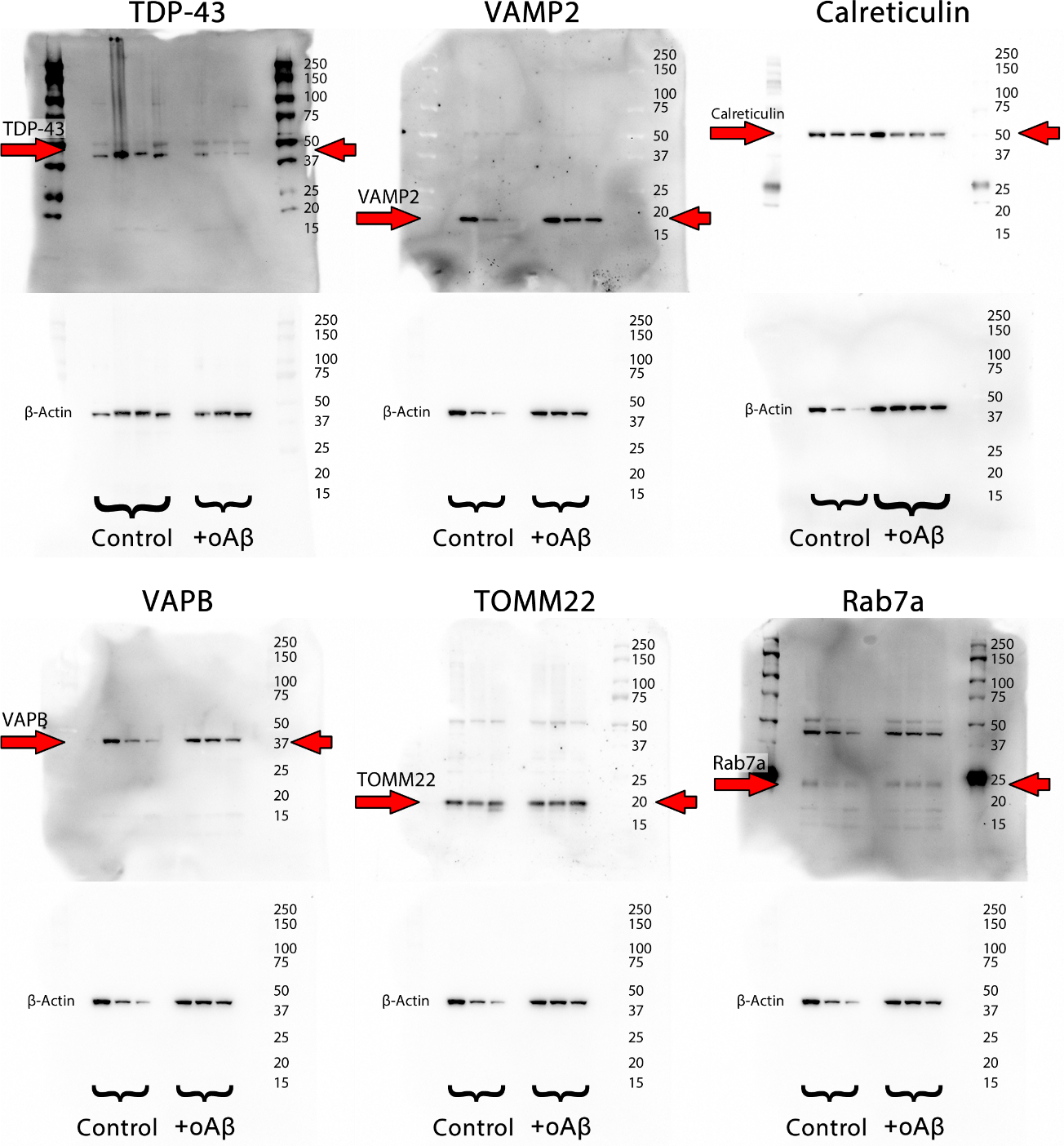

Supplementary Figure S3. Representative Western blots depicting AF22 protein expression changes in response to oAβ challenge. Densitometric quantifications are presented in Supplementary Figure S2. The images corresponding to VAMP2, VAPB, TOMM22 and Rab7a depict the same membrane that was stripped and re-probed with different primary antibodies.
